# Changes in gene expression linked with adult reproductive diapause in a northern malt fly species: a candidate gene microarray study

**DOI:** 10.1186/1472-6785-10-3

**Published:** 2010-02-01

**Authors:** Maaria Kankare, Tiina Salminen, Asta Laiho, Laura Vesala, Anneli Hoikkala

**Affiliations:** 1 Centre of Excellence in Evolutionary Research, Department of Biological and Environmental Science, P.O. Box 35, 40014 University of Jyväskylä, Finland; 2 Finnish DNA Microarray Centre, Bioinformatics team, Turku Centre for Biotechnology, Tykistökatu 6, FI-20521 Turku, Finland

## Abstract

**Background:**

Insect diapause is an important biological process which involves many life-history parameters important for survival and reproductive fitness at both individual and population level. *Drosophila montana*, a species of *D. virilis *group, has a profound photoperiodic reproductive diapause that enables the adult flies to survive through the harsh winter conditions of high latitudes and altitudes. We created a custom-made microarray for *D. montana *with 101 genes known to affect traits important in diapause, photoperiodism, reproductive behaviour, circadian clock and stress tolerance in model Drosophila species. This array gave us a chance to filter out genes showing expression changes during photoperiodic reproductive diapause in a species adapted to live in northern latitudes with high seasonal changes in environmental conditions.

**Results:**

Comparisons among diapausing, reproducing and young *D. montana *females revealed expression changes in 24 genes on microarray; for example in comparison between diapausing and reproducing females one gene (*Drosophila cold acclimation gene, Dca*) showed up-regulation and 15 genes showed down-regulation in diapausing females. Down-regulation of seven of these genes was specific to diapause state while in five genes the expression changes were linked with the age of the females rather than with their reproductive status. Also, qRT-PCR experiments confirmed *couch potato *(*cpo*) gene to be involved in diapause of *D. montana*.

**Conclusions:**

A candidate gene microarray proved to offer a practical and cost-effective way to trace genes that are likely to play an important role in photoperiodic reproductive diapause and further in adaptation to seasonally varying environmental conditions. The present study revealed two genes, *Dca *and *cpo*, whose role in photoperiodic diapause in *D. montana *is worth of studying in more details. Also, further studies using the candidate gene microarray with more specific experimental designs and target tissues may reveal additional genes with more restricted expression patterns.

## Background

Several insect species in temperate regions develop and reproduce during the summer, but become dormant as the winter approaches to avoid unfavourable conditions for development and reproduction. This dormancy is usually linked with a period of developmental arrest, i.e. diapause, at the embryonic, larval, pupal or adult stage. Adult reproductive diapause, where oogenesis and vitellogenesis are arrested, has been studied in monarch butterflies [1] and in several grasshopper [2] and Drosophila species [e.g. [3]; see also [4]]. In *Drosophila *species, this kind of diapause has been found to offer resource allocation trade-offs: the females entering reproductive diapause early in the summer will not produce progeny during the ongoing season, but they have a higher stress resistance, age more slowly [5] and have better chances to survive over the winter period and produce progeny during the next summer than the non-diapausing females. In most organisms the onset of reproductive diapause is largely determined by the length of the light period (photoperiodic diapause), which is not surprising, as seasonal changes in the day length in different latitudes are more predictable and consistent than changes in temperature or humidity [4]. Photoperiodic diapause also allows the females to anticipate the coming cold season in advance, which is very important for organisms with a long generation time. From an evolutionary point of view, reproductive diapause is an important biological process which involves many life-history parameters important for survival and reproductive fitness at both individual and population levels.

The discovery of an adult photoperiodic reproductive diapause in *Drosophila melanogaster *[6,7] opened a possibility to study the role of the circadian clock genes in photoperiodic time measurement and diapause, as well as interactions between genes affecting photoperiodism and hormonal and physiological changes involved in diapause. The finding also gave a chance to search for genes affecting fitness-related traits linked with diapause, e.g. life span, age-specific mortality, fecundity, resistance to cold and starvation stress, lipid content, development time, egg-to adult viability, accessory gland synthesis and mating behaviour [8-10]. *D. melanogaster *females are known to enter adult reproductive diapause in response to short days and low temperatures in Europe [11] and in North America [10,12]. However, Emerson *et al*., [13] showed lately that in two natural populations representing latitudinal extremes in eastern North America temperature is the main determinant of the diapause. Even though the reproductive diapause in *D. melanogaster *seems to be a relatively weak response to photoperiod and is only observed at temperatures below 14°C [7,14], the genes found to be linked with sexual maturation and diapause in this species are good candidate genes for diapause studies in more northern Drosophila species with stronger, longer-lasting photoperiodic diapause responses.

Our study species, *D. montana*, belongs to the *D. virilis *group, which originated in continental Asia. It has expanded its distribution around the northern hemisphere from 30°N to 70°N in latitude and from 0 to 3000 meters in altitude, adapting to various kinds of environmental conditions [15,16]. The females of this species have the ability to survive through the harsh winter conditions in photoperiodic adult reproductive diapause [17]. They have adapted to environmental conditions in northern Scandinavia with a mean temperature of less than 0°C for up to six winter months, a minimum temperature reaching occasionally -30°C or even below. *D. montana *flies predict the forthcoming cold season mainly by changes in the day length even though also the temperature seems to have some effect on the percentage of diapausing females [18].

We had two aims in this study. The first aim was to create a custom-made microarray for *D. montana *with candidate genes known to play a role in traits linked with reproductive diapause in *D*. *melanogaster*. The second aim was to determine which of the candidate genes show expression changes, and in which direction, in comparisons among diapausing (i.e. those in reproductive diapause), reproducing and young *D. montana *females. Our candidate gene microarray proved to be a practical and cost-effective way to use information on gene function obtained largely through mutagenesis in a genetic model species and to study whether the same genes play a role in adaptation to different environmental conditions in an ecologically interesting non-model organism. Out of the 101 genes assayed, 24 showed differences in their expression patterns in comparisons among *D. montana *females at different reproductive modes.

## Methods

### Study flies

We used *D. montana *flies from an isofemale line O3F66 originated from Oulanka (Northern Finland; 66°N) and maintained in the laboratory under diapause-preventing conditions (continuous light, 19°C) since summer 2003. In this isofemale line 98-100% of the female flies will reach sexual maturity (i.e., will not enter diapause) in continuous light (L:L) while in a light dark (L:D) cycle of 16:8 97% of them will enter reproductive diapause at 16°C (Salminen et al., unpublished). Female flies for the present study were collected under a light CO_2 _anaesthesia within one day after their emergence, i.e. before the photoperiodic induction of diapause occurred (Salminen *et al*., unpublished). One day old flies (three replicates, each including 13 flies) were transferred straight from the culturing conditions into liquid nitrogen for RNA extractions. The rest of the females were placed in vials (13-15 flies per vial) containing 7 ml of yeast-sucrose-agar medium [19] with about 5 mg of yeast sprinkled on top of the medium. The samples of reproducing females were obtained by maintaining the vials in a climate chamber in continuous light (24:0; diapause preventing conditions) for 14 days. Samples of diapausing females were obtained by keeping the vials in a climate chamber in a L:D cycle of 16:8 (diapause inducing conditions) for the same time period. The temperature in both chambers was 16 ± 1°C; lower temperatures were not used to avoid cold acclimation. Three sets of 13 females representing the two groups (reproducing/diapausing) were snap-frozen in liquid nitrogen immediately after collecting them from the climate chambers during a light period (two to three hours after lights went on in 16:8 light dark cycle). Flies were then stored at -84°C for RNA extractions.

### Designing a candidate gene DNA microarray for D. montana

The species-specific candidate gene microarray constructed for *D. montana *included 101 genes (Additional file 1) known to affect diapause (7 genes), phototransduction (25 genes), courtship behaviour (14 genes) and circadian clock (26 genes), as well as responses to cold (2 genes) and heat (27 genes) in *D. melanogaster*. The genes were selected from the *D. melanogaster *genome [20] using FlyBase gene ontology searches on biological response [21]. The most conserved regions of the candidate genes were indentified by aligning the exon regions of the *D. melanogaster *gene to their orthologs in *D. virilis *with Blat search in UCSC Genome Bioinformatics database [22]. *D. virilis *was chosen as a reference species, as it belongs to the same species group as *D. montana *and has its whole genome sequenced (Agencourt Bioscience Corporation).

The primers for the candidate genes of *D. montana *were designed for one to three unique 200-500 bp regions with Primer3 program [23] or manually, using information on the gene alignments mentioned above (primer sequences are available from the authors upon request). The gene sequences of *D. montana *were amplified with PCR and sequenced using both DNA and cDNA as a template to observe possible intron regions. The sequenced gene regions of *D. montana *were checked against *D. melanogaster *annotated genes and the genome sequences of *D. melanogaster *and *D. virilis *in FlyBase to ensure that they represented right genes and did not have homology to any other regions of the genome. Next, an online program e-Array 5.3 (Agilent) was used to design one to four 60 bp oligonucleotide probes per gene using exon regions. Finally, the probes were synthesized in situ with liquid chemistry and arrayed on an "Agilent 60-mer Multi-Pack Gene Expression Microarray" platform with one-colour system.

### Preparing the RNA samples for the microarray

The three samples for the microarray experiments included young females ("young"), 14 days old females cultured under diapause-preventing ("reproducing") and 14 days old females cultured under diapause-inducing conditions ("diapausing"). Total RNA was extracted from the whole body of 13 individuals per sample with three replicates per sample. Qiagen RNA extraction kit with RNase-Free DNase treatment was used according to the manufacturer's protocols (Qiagen). The RNA was purified using Qiagen RNAeasy purification kit and the concentration and the purity of each sample was measured with NanoDrop ND-1000 spectrophotometer (NanoDrop Technologies, Wilmington, DE, USA). 350 ng of total RNA from each sample was amplified and Cy3-labeled with Agilent's Low RNA Input Linear Amplification Kit PLUS (One Color) and processed together with Agilent's One-Colour RNA Spike-in Kit. The RNA/cRNA concentration (and also specific activity for cRNA) was checked with Nanodrop ND-1000 and the RNA/cRNA quality with BioRad's Experion electrophoresis station, both before and after the amplifications. 600 ng of each cRNA sample was hybridized to Agilent's 8 × 15K *D. montana *custom arrays with Gasket slide at 65°C overnight (17 h) using Agilent's Gene Expression Hybridization Kit and washed with Agilent's Gene Expression Wash Pack and Stabilization and Drying solution. Arrays were scanned with Agilent Technologies Scanner (model G2505B) and numerical results were extracted with Feature Extraction version 9.5.3.1 using 018413_D_F_20071204 grid, GE1-v5_95_Feb07 protocol and GE1_QCM_Feb07 QC metric set.

In addition to the 101 candidate genes we placed on our chip, we used seven genes, *Ef1α48D, eIF-4a, Gapdh1, Gapdh2, RpL11, RpL19 *and *RpL27A*, which have been used as control genes in *Drosophila *whole genome microarrays (GeneChip Drosophila genome 2.0 Array, Affymetrix, 2006). These genes were added on the chip to select the best control genes for microarray validation with qRT-PCR analysis.

### Statistical analysis

The microarray contained 197 unique probe sequences representing 101 candidate genes and seven housekeeping genes. Some of the probes had two copies per array, in which case the averages of their signals were calculated. The Agilent control probes included 77 negative control probes and 200 spike-in probes (10 different probe types, each represented by 20 copies per array). The raw images of the microarray were processed with Agilent's Feature Extraction software and further analyzed using R statistical analysis software (R Foundation for Statistical Computing) and related Bioconductor analysis package [24].

Data from small custom arrays cannot be normalized using traditional normalization methods developed for the whole genome arrays, because the common assumption of an unbiased trend of up and down-regulated genes and on the modulation of a small proportion of genes may not hold. There are, however, several alternative methods for small custom arrays [see [25]]. Variance stabilizing normalization method (VSN, [26]) using spike-in controls performs best with the small data sets, as it can be parameterized to allow up to 50% of the genes to be modulated [27]. In our data set this allowed all the experimental probes (representing about half of the probes) to be modulated between sample groups (the spike-in probes not expected to modulate formed the second half). Accordingly, microarray data were normalized within the groups and across all samples using the VSN method. The statistical analyses were carried out using Bioconductor related Limma package [24] and the differentially expressed genes in each comparison were selected requiring a strict selection criteria of fold-change > 2.0 and false discovery rate (fdr) < 0.05. The false discovery rates were calculated using the Benjamini and Hochberg's method as implemented in the Limma package.

### Quantitative real time PCR (qRT-PCR)

Expression changes in six of the 24 genes that showed up- or downregulation on the microarray were validated with qRT-PCR. The genes represent all the main categories based on their function in *D. melanogaster *(diapause, phototransduction, courtship behaviour, circadian clock, and cold and heat tolerance, Additional file 1). Primers for these genes (Table 1) were the same as those used in the microarray experiment, except in the case of *Dca *and *cpo *genes for which the original PCR product was too long to be used in qRT-PCR analysis. Samples of diapausing, reproducing and young females were collected as described earlier and cDNA was generated by reverse transcription with iScript cDNA kit (Bio-Rad) from newly made RNA extractions. RNA was exracted as described earlier and the purity of each sample was measured with NanoDrop ND-1000 spectrophotometer (NanoDrop Technologies, Wilmington, DE, USA). *Ef1α48D*(*Elongation factor 1 aplha 48D*) was used as reference gene for comparisons of diapausing and reproducing flies and *eIF-4a *(*Eukaryotic initiation factor*4a) gene was used in comparison of diapausing or reproducing females to young females. These genes were selected on the basis of their expression stability among all the control genes (i.e. housekeeping genes) tested in the microarray analysis. Amplification efficiency of each primer pair was checked with serial dilutions of cDNA (data not shown) and the real efficiency values (all with E-values > 90% and R^2^-values > 0.980) were used in the analysis. Real time PCR analysis was performed in 25 μl reactions containing SYBR Green SuperMix (BioRad), 0.3 μM of each gene-specific primer and 1 μl of cDNA solution. Cycling conditions in BioRad CFX96 instrument were 95°C for 3 min, followed by 40 cycles of denaturing at 95°C for 10 sec and annealing at 54°C for 30 sec and extension at 72°C for 30 sec. Fold change relative to the expression of the sample genes and reference genes was calculated using normalized expression (ΔΔ(Ct)) method with default threshold values using CFX Manager Software which will give the same result as the Pfaffl method [28] when using one target and one reference gene [29]. Two to three biological replicates (each mixture of 13 individual flies) were analyzed in each of the comparisons and the purity of the PCR reactions was checked with the melting curve analysis after amplification. Independent-samples t-test (SPSS 13 for windows, SPSS Inc.) was used to test the statistical significance of normalized gene expression in different comparisons.

**Table 1:** Primer pairs used in the qRT-PCR. See text for details in primer design

Gene ID	Biological function	Primers (5' - 3')
*cpo*	Diapause	5' - AGTCACAGTCGCACGAGTCAA - 3' 5' - TTCACGGAGTCCATGCTCTGT - 3'
*FKBP59*	Phototransduction	5' - GCTCCAAACTACGCTTACGG - 3' 5' - CGCTTTTTATCGCTTGGTTC - 3'
*tilB*	Courtship behavior	5' - GTCGCGATCTCAAGATCCT - 3' 5' - TGCGGATAATCGACACACG - 3'
*Fmr1*	Circadian rhythm	5' - CCATTCTTGCCAATCACCTT - 3' 5' - GTGTGCGCGAAGATCTCAT - 3'
*Dca*	Cold tolerance	5' - CAGAACAAGACGTACAGGAC - 3' 5' - TCATCGCCAATGTAGCGCAT - 3'
*Hsp26*	Heat tolerance	5' - CATCGGAGGACAGAGAGGAG - 3' 5' - ATTGTCCACCACCTTGACGT - 3'

## Results

The microarray protocol used in the present study proved to be both reliable and repeatable. Experimental probe intensities of the raw data on the microarray were highly correlated between the three replicates of each of the three female samples (median Pearson's correlation = 0.885). Correlations between the 'technical replicates' (RNA from three samples of diapausing females hybridized on arrays at two different hybridization times) were even higher (Pearson's correlations > 0.990), indicating high repeatability and low technical variation. The microarray data has been deposited in NCBI's Gene Expression Omnibus [30] and are accessible through GEO Series accession number GSE18475 and GPL accession number GPL9293.

### Up- and down-regulation of candidate genes in microarray

**Figure 1:**
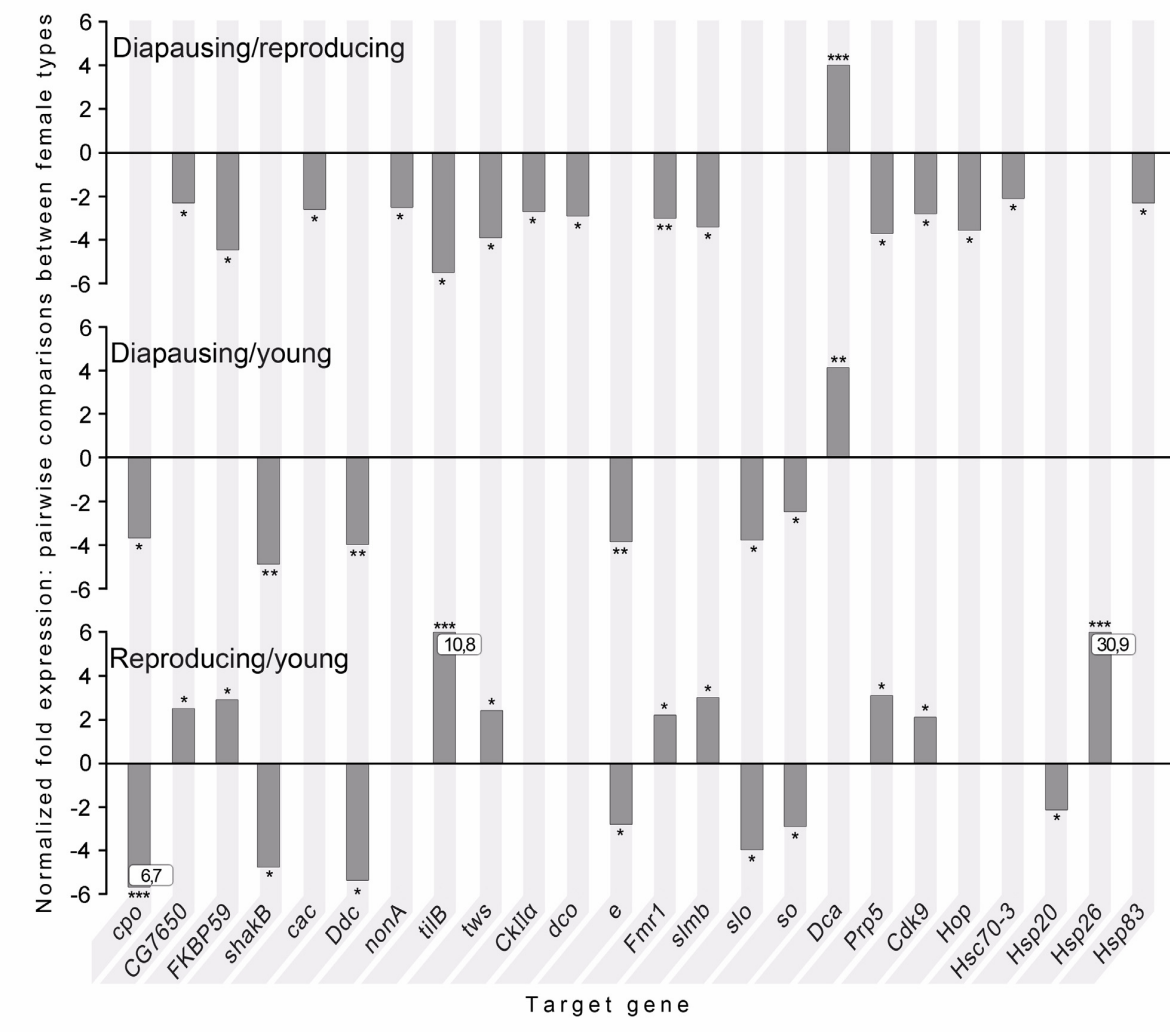
**Normalized fold expression of the candidate genes in microarray analysis**. Normalized fold expression of the candidate genes in diapausing females compared to reproducing females, in diapausing females compared to young females and in reproducing females compared to young females in microarray analysis. Up-regulation is shown with the positive values and down-regulation with the negative values on y-axis. Significance levels: *0.05 > P > 0.01, **0.01 > P > 0.001, ***P < 0.001. In *cpo *gene down-regulation and in *tilB *and *Hsp26 *genes up-regulation was more than 6-fold and is indicated with numbers in the boxes.

Comparison between diapausing, reproducing and young *D. montana *females revealed significant expression changes in 24 of the 101 candidate genes on the microarray (Fig. 1). We were most interested in genes showing differential expression in diapausing females versus reproducing females. In this comparison the diapausing females showed up-regulation in only one gene, *Drosophila cold acclimation *gene (*Dca*), and down-regulation in 15 genes. Down-regulation of seven of the genes (*cac, nonA, CkIIα, dco, Hop, Hsc70-3, Hsp83*) was specific to diapause state (they only appeared in the comparison between diapausing and reproducing females) while in five genes (*shakB, Ddc, e, slo, so*) the expression changes were linked with the age of the females rather than with their reproductive status (Fig. 1). One additional gene, *couch potato *(*cpo*), which is known to affect diapause in *D. melanogaster *(see Discussion), showed a significant down-regulation in diapausing and reproducing females compared to young females and a marginally significant (FC = 1.8 and p = 0.055) up-regulation in diapausing females compared to reproducing females. Eigth of the 15 genes *(CG7650, FKBP59, tilB, tws, Fmr1, slmb, Prp5, Cdk9) *that were down-regulated in diapausing females when compared to reproducing females showed down-regulation also in young females when compared to reproducing females. The last two genes, *Hsp20 *and *Hsp26*, were expressed only when reproducing females were compared to young females (Fig. 1).

### Confirmation of microarray results with qRT-PCR

Changes in the function of six genes, *cpo *(diapause), *FKBP59 *(phototransduction), *tilB* (courtship behaviour), *Fmr1 *(circadian clock), *Dca* (cold tolerance) and *Hsp26 *(heat tolerance), showing differential expression between at least two of the three female developmental stages in the microarray analysis were confirmed with qRT-PCR (we made altogether 12 comparisons). All the tested genes showed expression changes in the same direction in qRT-PCR as in the microarray analysis, even though the magnitude and statistical significance of changes differed between the two methods in half of the cases (Additional file 2). Comparisons between the diapausing and reproducing females showed significant up-regulation in diapausing females in *cpo *and *Dca *and down-regulation in *tilB*, while in *Fmr1 *and *FKBP59 *the expression difference remained non-significant (Additional file 2, Fig. 2). In the case of *cpo *it is worth to note that qRT-PCR revealed significant differences both in comparisons of diapausing and reproducing and reproducing and young females while in microarray analysis the difference between diapausing and reproducing females was marginally non-significant (p = 0.055).

**Figure 2:**
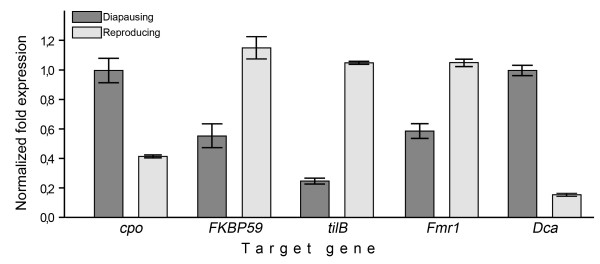
**Normalized fold expressions of candidate genes in qRT-PCR analysis**. Normalized fold expressions and standard deviations of *cpo, CG7650, tilB, Fmr1 *and *Dca *genes in diapausing and reproducing females in qRT-PCR analysis.

## Discussion

### Feasibility of a candidate gene array for detecting evolutionary interesting genes in non-model organisms

Gene expression studies have previously been performed for genetically less well-known *Drosophila *species using microarrays constructed for *D. melanogaster*, but due to high levels of genetic divergence between *D. melanogaster *and the species under investigation (usually several My), these kinds of data may be difficult to interpret and may lack reliability [e.g. [31]]. Another solution with non-model organisms is to prepare custom microarrays or to trace changes in gene expression via transcriptome analyses [e.g. [32]]. Both of these methods are feasible, but they are still quite expensive and difficult to analyze when genome information is incomplete. We decided to create a candidate gene microarray for *D. montana *to survey the genes underlying the life-history traits important in reproductive diapause in a model Drosophila species. The used multi-pack gene expression microarray with one-colour system provides a possibility to compare several (up to eight) samples with each other on one chip, and thus is cost-effective and appropriate for ecological study designs. The most substantial restriction in a candidate gene microarray is that it gives information only on a limited number of genes. However, new genes can be added to new versions of the array once detected in model organisms or found in the study species e.g. through transcriptome or QTL analyses. Recently published work on gene expression during *D. melanogaster *egg development before and after reproductive diapause indicated several genes connected to diapause behaviour [33] worth of adding to our array.

### Changes in gene expression pattern linked with reproductive diapause

Emerson *et al*. have argued that the incidence of diapause is determined by genes in three successive modules, photoperiodism, hormonal events and the diapause itself and an extra module, circadian clock, which can potentially affect diapause through any of the other three [34]. The present study design has allowed us to detect gene expression changes mainly in the hormonal and diapause modules.

The most interesting findings in our study were the up-regulation of *Dca *gene in diapausing females when compared to reproducing and young females and the expression changes in *cpo *gene between the three female types. *Dca *(also called *smp-30*, according to its mammalian homologue) has been suggested to be associated with Ca^2+ ^regulation [35], and it may play a role in maintaining Ca^2+ ^content during cold exposure [36]. In *D. melanogaster *the expression level of this gene has been found to increase during 1-2 day cold acclimation in 15°C [36] and to decrease during recovery after cold treatment in 0°C [37,38]. Expression level of *Dca *has also been found to increase with age in *D. melanogaster *[36], contrary to the situation in some mammal species [35]. In our study, the high expression level of *Dca *in diapausing females may be connected to female cold tolerance, which is known to increase during diapause in *D. montana *(L. Vesala, unpublished). The fact that *Dca *showed no expression changes between the reproducing and young females suggests that the detected transcriptional changes may not be connected to fly aging. The *cpo *gene has been suggested to play an important role in diapause in *D. melanogaster *[e.g. [39]]. This gene could provide a link between the insulin signaling pathway and the downstream hormones involved directly in the regulation of vitellogenesis [34]. *cpo *is expressed in the peripheral and central nervous system of *Drosophila *embryos, larvae, and adults [40] and was originally named "couch potato" because several partial loss-of-function alleles of this gene cause hyperactive behavior in *D. melanogaster *[40]. *cpo *is also known to be involved in the latitudinal cline of diapause behavior in *D. melanogaster *[39]. In our study, *cpo *was highly down-regulated in diapausing and reproducing females, when compared to young females and it also showed significant up-regulation in diapausing females when compared to reproducing females in qRT-PCR analysis (in microarray analysis the last comparison gave a marginally significant p-value). These findings suggest that *cpo *could play a role in diapause in *D. montana*.

Another interesting group of genes in the scope of the present study are the seven genes down-regulated in diapausing females, but not in young females, when compared to reproducing females. Two of these genes, *Casein kinase alpha II subunit *(*CkIIα*) and *discs overgrown* (*dco*) have been listed as circadian rhythm genes in FlyBase (see Additional file 1) and they may be involved in several biological processes such as locomotor activity or mating behavior [41], which might explain their low expression in diapausing females. Two genes, *no on or off transient-A *(*nonA*) and *cacophony *(*cac*), have been found to affect courtship behavior and male courtship song in *D. melanogaster*[42]; *nonA *gene is also known to regulate other genes and have pleiotropic effects on fly vision [43], whereas *cac *is known to be involved in adult locomotor behavior [44], phototransduction and visual behavior [45]. Finally, the three last genes down-regulated only in diapausing females were the heat shock protein genes *Hsp70/Hsp90 organizing protein homolog *(*Hop*), *Heat shock protein cognate 3* (*Hsc70-3*)and *Heat shock protein 83 *(*Hsp83*). Some studies have indicated that changes in the expression level of heat shock genes could be connected with insect diapause, but the data are not congruent [46,47]. It is worth noting that while other heat shock genes in our study showed significant down-regulation in diapausing females, *Heat shock protein 20*(*Hsp20*)and *Heat shock protein 26 *(*Hsp26*) showed expression changes only between reproducing and young females (*Hsp20 *was down-regulated and *Hsp26 *up-regulated in reproducing females when compared to young females).

Some genes that showed expression variation on our array may not play an important role in the regulation of reproductive diapause itself, but instead may exhibit pleiotropic effects on life-history traits associated with diapause. On the other hand, some genes like the insulin-pathway genes (*Dp110*)and *Insulin-like receptor*(*InR*) did not show expression changes on our array, even though insulin signaling has been shown to play a role in the diapause in nematodes [48], mosquitoes [49] and Drosophila [50]. In particular, *Dp110 *gene has been shown to be necessary for ovarian development of *D. melanogaster *and allelic variation in this gene has been found to be associated with latitudinal increase in the incidence of diapause [50]. Also some genes important in photoperiodic time measurement, like *period *(*per*) and *timeless *(*tim*), were lacking from the list of genes showing expression variation in our study. Expression changes in these genes need to be studied with specific experimental design, taking into account e.g. the daily cycles in the expression level of the genes and targeting the studies on specific tissues like the heads of the flies.

## Conclusions

The present study shows that a candidate gene microarray provides a practical and cost-effective platform for detecting genes that play a role e.g. in adaptation into different kinds of environmental conditions in ecologically interesting but genetically less well-known organisms. The value of this kind of arrays depends on how well the chosen candidate genes represent the genes and genetic pathways affecting the traits of interest, how repeatable the results are and how well the experimental design supports the asked questions. New genes can be added on array once detected in other species (see [33]) or found in the study species e.g. through transcriptome analyses. Candidate gene arrays will be especially useful when combined with other methods of the candidate gene approach; using SNPs of the array genes as markers in association studies will, for example, help to trace the link between phenotype, genotype and gene expression. The genes that show expression changes on array, like *Dca *and *cpo *in the present study, will be good targets of future studies on molecular evolution. Since traits like diapause incidence vary between populations, especially along latitudinal clines, the frequency of any putative mutation changing the function of genes affecting these traits would be predicted to show variation linked with phenotypic variation. Also, it will be interesting to trace signs of selection on the coding region of the genes.

## Authors' contributions

MK constructed the microarray chip, analyzed the results, performed the qRT-PCR analysis, and wrote the manuscript. TS participated in the microarray construction, study design and manuscript writing and cultured the flies and extracted the RNA for the microarray study. AL performed the statistical analysis for microarray results and participated in the manuscript writing. LV made the RNA extractions for the qRT-PCR analysis and participated in the manuscript writing. AH participated in the design of the study and coordination and helped to write the manuscript. All authors read and approved the final manuscript.

## Supplementary Material

Additional file 1 **Candidate and housekeeping genes on a species-specific microarray constructed for *D. montana***. Candidate and housekeeping genes on a species-specific microarray constructed for *D. montana *presented in alphabetical order. Information of biological processes of the genes is collected from FlyBase database (FB2009_04), released in April 27, 2009. Housekeeping genes are marked with asterisks. Click here for file

Additional file 2 **Fold changes in gene expression in microarray/qRT-PCR analysis: comparisons between the three female groups**. Fold changes in gene expression of selected candidate genes in microarray and qRT-PCR analysis. Comparisons were made between diapausing, reproducing and young females. Significance levels are indicated as follows: ^NS^Non-significant, *0.05 > P > 0.01, **0.01 > P > 0.001, ***P < 0.001. Click here for file

## References

[B1] Herman WS Studies on the reproductive diapause of the monarch butterfly *Danaus plexippus* Biol Bull 1981 160 89 106 10.2307/1540903

[B2] Broza M Pener MP Hormonal control of the reproductive diapause in the grasshopper, *Oedipoda miniata* CMLS 1969 25 414 415 10.1007/BF01899955 5799241

[B3] Kimura MT Geographic variation of reproductive diapause in the *Drosophila auraria *complex (Diptera: Drosophilidae) Physiol Entomol 1984 9 425 431 10.1111/j.1365-3032.1984.tb00784.x

[B4] Tatar M Yin C-M Slow aging during insect reproductive diapause: why butterflies, grasshoppers and flies are like forms Exp Gerontol 2001 36 723 738 10.1016/S0531-5565(00)00238-2 11295511

[B5] Tatar M Chien SA Priest NK Negligible Senescence during Reproductive Dormancy in *Drosophila melanogaster* Am Nat 2001 158 1 11 10.1086/321320 18707322

[B6] Saunders DS The Circadian Basis of Ovarian Diapause Regulation in *Drosophila melanogaster *: Is the period Gene Causally Involved in Photoperiodic Time Measurement J Biol Rhythms 1990 5 315 331 10.1177/074873049000500404 2133139

[B7] Saunders DS Henrich VC Gilbert LI Induction of diapause in *Drosophila melanogaster*: photoperiodic regulation and impact of arrhythmic clock mutations on time measurement Proc Natl Acad Sci USA 1989 86 3748 3752 10.1073/pnas.86.10.3748 2498875 PMC287217

[B8] Tauber MJ Tauber CA Masaki S Seasonal adaptations of Insects 1986 Oxford University Press, New York

[B9] Schmidt PS Matzkin L Ippolito M Eanes WF Geographic variation in diapause incidence, life-history traits, and climatic adaptation in *Drosophila melanogaster* Evolution 2005 59 1721 1732 16331839

[B10] Schmidt PS Paaby AB Heschel MS Genetic variance for diapause expression and associated life histories in *Drosophila melanogaster* Evolution 2005 59 2616 2625 16526509

[B11] Tauber E Zordan M Sandrelli F Pegoraro M Osterwalder N Breda C Daga A Selmin A Monger K Benna C Rosato E Kyriacou C Costa R Sokolowski W Natural Selection Favors a Newly Derived timeless Allele in *Drosophila melanogaster* Science 2007 316 1895 1898 10.1126/science.1138412 17600215

[B12] Williams KD Sokolowski MB Diapause in *Drosophila melanogaster *females: a genetic analysis Heredity 1993 71 312 317 10.1038/hdy.1993.141 8407357

[B13] Emerson KJ Uyemura AM McDaniel KL Schmidt PS Bradshaw WE Holzapfel CM Environmental control of ovarian dormancy in natural populations of *Drosophila melanogaster* J Comp Physiol A 2009 195 825 829 10.1007/s00359-009-0460-5 19669646

[B14] Saunders D Gilbert LI Regulation of ovarian diapause in the fruit fly *Drosophila melanogaster *by photoperiod at moderately low temperature J Insect Physiol 1990 36 95 200 10.1016/0022-1910(90)90122-V

[B15] Mirol PM Schäfer MA Orsini L Routtu J Schlötterer C Hoikkala A Butlin RK Contrasting phylogeographic patterns in *Drosophila virilis *and *Drosophila montana* Mol Ecol 2007 16 1085 1097 10.1111/j.1365-294X.2006.03215.x 17305862

[B16] Throckmorton LH Carson HL Thompson JN Jr Ashburner M The virilis species group The Genetics and Biology of Drosophila 1982 3b Academic Press, London

[B17] Lumme J Dingle H Phenology and Photoperiodic diapause in Northern Populations of Drosophila Evolution of Insects Migration and Diapause 1978 45 169

[B18] Watabe H Photoperiodic responses in *Drosophila virilis *species group (Diptera, Drosophilidae) from Japan Kontyu, Tokyo 1983 51 628 634

[B19] Rosato E Kyriacou CP Analysis of locomotor activity rhythms in Drosophila Nat Protoc 2006 1 559 568 10.1038/nprot.2006.79 17406282

[B20] Adams MD Celniker SE Holt RA Evans CA Gocayne JD Amanatides PG Scherer SE Li PW Hoskins RA Galle RF The genome sequence of *Drosophila melanogaster* Science 2000 287 2185 2195 10.1126/science.287.5461.2185 10731132

[B21] FlyBase http://flybase.org

[B22] UCSC Genome Bioinformatics database http://genome.ucsc.edu

[B23] Primer3 program http://frodo.wi.mit.edu/primer3

[B24] Gentleman RC Carey VJ Bates DM Bolstad B Dettling M Dudoit S Ellis B Gautier L Ge Y Gentry J Hornik K Hothorn T Huber W Iacus S Irizarry R Leisch F Li C Maechler M Rossini AJ Sawitzki G Smith C Smyth G Tierney L Yang JY Zhang J Bioconductor: open software development for computational biology and bioinformatics Genome Biol 2004 5 R80 10.1186/gb-2004-5-10-r80 15461798 PMC545600

[B25] Oshlack A Emslie D Corcoran LM Smyth GK Normalization of boutique two-color microarrays with a high proportion of differentially expressed probes Genome Biol 2007 8 1 R2 10.1186/gb-2007-8-1-r2 17204140 PMC1839120

[B26] Huber W von Heydebreck A Sültmann H Poustka A Vingron M Variance stabilization applied to microarray data calibration and to the quantification of differential expression Bioinformatics 2002 18 96 104 10.1093/bioinformatics/18.suppl_1.s96 12169536

[B27] Davison TS Johnson CD Andruss BF Analyzing micro-RNA expression using microarrays Methods Enzymol 2006 411 14 34 10.1016/S0076-6879(06)11002-2 16939783

[B28] Pfaffl MW A new mathematical model for relative quantification in real-time RT-PCR Nucleic Acids Res 2001 29 e45 10.1093/nar/29.9.e45 11328886 PMC55695

[B29] CFX Manager™ Software Help Resources Bio-Rad Laboratories 2007

[B30] Gene Expression Omnibus (GEO) database https://www.ncbi.nlm.nih.gov/geo

[B31] Nuzhdin SV Wayne ML Harmon KL McIntyre LM Common pattern of evolution of gene expression level and protein sequence in Drosophila Mol Biol Evol 2004 21 1308 1317 10.1093/molbev/msh128 15034135

[B32] Vera JC Wheat CW Fescemyer HW Frilander MJ Crawford DL Hanski I Marden JH Rapid transcriptome characterization for a nonmodel organism using 454 pyrosequencing Mol Ecol 2008 17 1636 47 10.1111/j.1365-294X.2008.03666.x 18266620

[B33] Baker DA Russell S Gene expression during *Drosophila melanogaster *egg development before and after reproductive diapause BMC Genomics 2009 10 242 10.1186/1471-2164-10-242 19463195 PMC2700134

[B34] Emerson KJ Bradshaw WE Holzapfel CM Complications of complexity: integrating environmental, genetic and hormonal control of insect diapause Trends Genet 2009 25 217 225 10.1016/j.tig.2009.03.009 19375812

[B35] Fujita T Inoue H Kitamura T Sato N Shimosawa T Maruyama N Senescence marker protein-30 (SMP30) rescues cell death by enhancing plasma membrane Ca^2+^-pumping activity in Hep G2 cells Biochem Biophys Res Comm 1998 250 374 380 10.1006/bbrc.1998.9327 9753637

[B36] Goto SG Expression of Drosophila homologue of senescence marker protein -30 during cold acclimation J Insect Physiol 2000 46 1111 1120 10.1016/S0022-1910(99)00221-8 10817837

[B37] Qin W Neal SJ Robertson RM Westwood JT Walker VK Cold hardening and transcriptional change in *Drosophila melanogaster* Insect Mol Biol 2005 14 6 607 613 10.1111/j.1365-2583.2005.00589.x 16313561

[B38] Sinclair BJ Gibbs AG Roberts SP Gene transcription during exposure to, and recovery from, cold and desiccation stress in *Drosophila melanogaster* Insect Mol Biol 2007 16 435 443 10.1111/j.1365-2583.2007.00739.x 17506850

[B39] Schmidt PS Zhu CT Das J Batavia M Yang L Eanes MF An amino acid polymorphism in the couch potato gene forms the basis for climatic adaptation in *Drosophila melanogaster* Proc Natl Acad Sci USA 2008 105 16207 16211 10.1073/pnas.0805485105 18852464 PMC2570987

[B40] Bellen HJ Vaessin H Bier E Kolodkin A D'Evelyn D Kooyer S Jan YN The Drosophila *couch potato *gene: an essential gene required for normal adult behavior Genetics 1992 131 365 375 1644278 10.1093/genetics/131.2.365 PMC1205011

[B41] Mackay TFC Heinsohn SL Lyman RF Moehring AJ Morgan TJ Rollman SM Genetics and genomics of Drosophila mating behavior Proc Natl Acad Sci USA 2005 102 6622 6629 10.1073/pnas.0501986102 15851659 PMC1131870

[B42] Peixoto AA Hall JC Analysis of temperature-sensitive mutants reveals new genes involved in the courtship song of Drosophila Genetics 1998 148 827 38 9504928 10.1093/genetics/148.2.827 PMC1459814

[B43] Rendahl KG Jones KR Kulkarni SJ Bagully SH Hall JC The *dissonance *nutation at the *no-on-transient A *locus of *D. melanogaster *: genetic control of courtship song and visual behaviors by a protein with putative RNA-binding motifs J Neurosci 1992 12 390 407 1740687 10.1523/JNEUROSCI.12-02-00390.1992 PMC6575613

[B44] Dellinger B Felling R Ordway RW Genetic modifiers of the Drosophila NSF mutant, comatose, include a temperature-sensitive paralytic allele of the calcium channel alpha1-subunit gene, *cacophony* Genetics 2000 155 203 211 10790395 10.1093/genetics/155.1.203 PMC1461054

[B45] Sokolowski MB Drosophila: *Genetics *meets behaviour Nat Rev Genet 2001 2 879 890 10.1038/35098592 11715043

[B46] Denlinger DL Regulation of diapause Annu Rev Entomol 2002 47 93 122 10.1146/annurev.ento.47.091201.145137 11729070

[B47] Goto SG Kimura MT Heat-shock-responsive genes are not involved in the adult diapause of *Drosophila triauraria* Gene 2004 326 117 122 10.1016/j.gene.2003.10.017 14729269

[B48] Fielenbach N Antebi A *C. elegans *dauer formation and the molecular basis of plasticity Genes Dev 2008 22 2149 2165 10.1101/gad.1701508 18708575 PMC2735354

[B49] Sim C Denlinger DL Insuling signaling and FOXO regulate the overwintering diapause of the mosquito *Culex pipiens* Proc Natl Acad Sci USA 2008 105 6777 6781 10.1073/pnas.0802067105 18448677 PMC2373331

[B50] Williams KD Busto M Suster ML So AK Ben-Shahar Y Leevers SJ Sokolowski MB Natural variation in *Drosophila melanogaster *diapause due to the insulin-regulated PI3-kinase Proc Natl Acad Sci USA 2006 103 15911 15915 10.1073/pnas.0604592103 17043223 PMC1635102

