# Supplementary material for: Changes in gene expression linked with adult reproductive diapause in a northern malt fly species: a candidate gene microarray study
Source: BMC Ecol. 2010 Feb 1;10:3. doi: 10.1186/1472-6785-10-3 (PMC2822739; doi:10.1186/1472-6785-10-3)
Supplement: Additional file 1 — Candidate and housekeeping genes on a species-specific microarray constructed for D. montana. Candidate and housekeeping genes on a species-specific microarray constructed for D. montana presented in alphabetical order. Information of biological processes of the genes is collected from FlyBase database (FB2009_04), released in April 27, 2009. Housekeeping genes are marked with asterisks. [file 1472-6785-10-3-S1.DOC]

**Additional file 1.** Candidate and housekeeping genes on a species-specific microarray constructed for *D. montana*.

| No. | Gene ID | Gene name/synonym | CG number | Biological processes where the gene is involved | Functional basis for selecting the gene |
| --- | --- | --- | --- | --- | --- |
| 1 | *Adar* | *Adenosine deaminase acting on RNA* | 12598 | Response to hypoxia, locomotor activity | Heat tolerance |
| 2 | *Adh* | *Alcohol dehydrogenase* | 3481 | Ethanol oxidation, behavioral response to ethanol | Diapause |
| 3 | *Ankyrin* | *Ank* | 1651 | Cytoskeletal anchoring at plasma membrane | Phototransduction |
| 4 | *ari-1* | *ariadne* | 5659 | Not known | Courtship behavior |
| 5 | *Arr1* | *Arrestin 1* | 5711 | Deactivation of rhodopsin mediated signaling | Phototransduction |
| 6 | *Arr2* | *Arrestin 2* | 5962 | Deactivation of rhodopsin mediated signaling | Phototransduction |
| 7 | *ato* | *atonal* | 7508 | Anatomical structure development | Courtship behavior |
| 8 | *b* | *black* | 7811 | Visual behavior | Phototransduction |
| 9 | *btv* | *beethoven* | 15148 | Microtubule-based movement, | Courtship behavior |
| 10 | *cac* | *cacophony* | 1522 | Courtship behavior, adult locomotory behavior | Courtship behavior |
| 11 | *Cam* | *Calmodulin* | 8472 | Kinetochore organization | Phototransduction |
| 12 | *CaMKII* | *Calsium/calmodulin-depend.prot.kin.II* | 18069 | Male courtship behavior, learning or memory | Phototransduction |
| 13 | *Cdc37* | *Cdc37* | 12019 | Protein kinase cascade | Heat tolerance |
| 14 | *Cdk9* | *Cyclin-dependent kinase 9* | 5197 | RNA elongation from RNA polymerase II promoter | Heat tolerance |
| 15 | *CdsA* | *CDP diglyceride synthetase* | 7962 | Terminal branching, open tracheal system | Phototransduction |
| 16 | *CG12020* | *Heat shock prot.cognate DnaJ, C-term.* | 12020 | Response to heat, protein folding | Heat tolerance |
| 17 | *CG14650* | *Heat shock prot.cognate DnaJ, N-term.* | 14650 | Response to heat, protein folding | Heat tolerance |
| 18 | *CG4049* | *CG4049* | 4049 | DNA repair | Heat tolerance |
| 19 | *CG4164* | *CG4164* | 4164 | Response to heat, protein folding | Heat tolerance |
| 20 | *CG5001* | *Heat shock prot.cognate DnaJ, N-term.* | 5001 | Response to heat, protein folding | Heat tolerance |
| 21 | *CG7650* | *CG7650* | 7650 | Phototransduction | Phototransduction |
| 22 | *CkIIalfa* | *Casein kinase II aplha subunit* | 17520 | Protein amino acid phosphoryl., locomotor rhythm | Circadian rhythm |
| 23 | *Clk* | *Clock* | 7391 | Biosynthetic process, sleep | Circadian rhythm |
| 24 | *CoI* | *Mitochondrial Cytochrome c oxidase I* | 34067 | Sleep, mitochondrial electron transport | Diapause |
| 25 | *cpo* | *couch potato* | 31243 | Synaptic transmission | Diapause |
| 26 | *CrebB17A* | *Cyclic-AMP resp.el.bind.prot. B17A* | 6103 | Regulation of transcription, circadian rhythm | Diapause |
| 27 | *dare* | *defective in the avoidance of repellents* | 12390 | Olfactory behavior, courtship behavior | Phototransduction |
| 28 | *Dat* | *Dopamine N acetyltransferase* | 3318 | Catecholamine metabolic process, sleep | Diapause |
| 29 | *Dca* | *Drosop. cold acclimation gene, smp-30* | 7390 | Not known | Cold tolerance |
| 30 | *dco* | *discs overgrown/double time* | 2048 | Regulation of biological process, sleep | Circadian rhythm |
| 31 | *Ddc* | *Dopa decarboxylase* | 10697 | Dopamine biosynthetic process from tyrosine | Courtship behavior |
| 32 | *disco* | *disconnected* | 9908 | Positive regul. of transcription, locomotor rhythm | Circadian rhythm |
| 33 | *DnaJ-1* | *DnaJ-like-1* | 10578 | Response to heat, protein folding | Heat tolerance |
| 34 | *dnc* | *dunce* | 32498 | Learning or memory, mating | Circadian rhythm |
| 35 | *dsf* | *dissatisfaction* | 9019 | Somatic sex determination, courtship behavior | Courtship behavior |
| 36 | *Dsor1* | *Downstream of raf1* | 15793 | Signal transduction | Circadian rhythm |
| 37 | *dy* | *dusky* | 9355 | Rhythmic behavior, circadian rhythm | Circadian rhythm |
| 38 | *e* | *ebony* | 3331 | Primary metabolic process | Circadian rhythm |
| 39 | *Ef1alfa48D** | *Elongation factor 1 alpha 48D* | 8280 | Translation elongation factor activity | Housekeeping gene |
| 40 | *eIF-4a** | *Eukaryotic initiation factor 4a* | 9075 | Translational initiation | Housekeeping gene |
| 41 | *FKBP59* | *FK506-binding protein FKBP59* | 4535 | Peripheral nervous system development | Phototransduction |
| 42 | *Fmr1* | *Fmr1* | 6203 | Phototransduction | Circadian rhythm |
| 43 | *fru* | *fruitless* | 14307 | Behavioral interaction between organisms | Courtship behavior |
| 44 | *Gapdh1** | *Glyceraldehyde 3 phosp.dehydrogen. 1* | 12055 | Glycolysis | Housekeeping gene |
| 45 | *Gapdh2** | *Glyceraldehyde 3 phosp.dehydrogen. 2* | 8893 | Glycolysis | Housekeeping gene |
| 46 | *Gbeta76C* | *G protein beta subunit 76C* | 8770 | Deactivation of rhodopsin mediated signaling | Phototransduction |
| 47 | *gl* | *glass* | 7672 | Response to red light | Circadian rhythm |
| 48 | *hang* | *hangover* | 32575 | Response to ethanol, response to heat | Heat tolerance |
| 49 | *Hop* | *Hsp70/Hsp90 organizing protein hom.* | 2720 | Protein folding | Heat tolerance |
| 50 | *Hsc70-2* | *Heat shock protein cognate 2* | 7756 | Protein folding, response to heat | Heat tolerance |
| 51 | *Hsc70-3* | *Heat shock protein cognate 3* | 4147 | Sleep, response to heat | Heat tolerance |
| 52 | *Hsc70-4* | *Heat shock protein cognate 4* | 4264 | Embryonic develop. via the syncytial blastoderm | Heat tolerance |
| 53 | *Hsc70-5* | *Heat shock protein cognate 5* | 8542 | Response to heat, protein folding | Heat tolerance |
| 54 | *Hsc70Cb* | *Hsc70Cb* | 6603 | Protein folding | Heat tolerance |
| 55 | *Hsf* | *Heat shock factor* | 5748 | Response to heat | Heat tolerance |
| 56 | *Hsp20* | *small heat shock protein hsp20 family* | 4461 | Response to heat | Heat tolerance |
| 57 | *Hsp26* | *Heat shock protein 26* | 4183 | Determination of adult life span, response to heat. | Heat tolerance |
| 58 | *Hsp60* | *Heat shock protein 60* | 12101 | Response to heat, protein folding | Heat tolerance |
| 59 | *Hsp67Bc* | *Heat shock gene 67Bc* | 4190 | Response to heat. | Heat tolerance |
| 60 | *Hsp68* | *Heat shock protein 68* | 5463 | Determination of adult life span, response to heat | Heat tolerance |
| 61 | *Hsp83* | *Heat shock protein 83* | 1242 | Cell cycle, anatomical structure development | Heat tolerance |
| 62 | *imd* | *immune deficiency* | 5576 | Immune response, defense response | Heat tolerance |
| 63 | *inaC* | *inactivation no afterpotential C* | 6518 | Adaptation of rhodopsin mediated signaling | Phototransduction |
| 64 | *inaD* | *inactivation no afterpotential D* | 3504 | Deactivation of rhodopsin mediated signaling | Phototransduction |
| 65 | *inaF* | *inactivation no afterpotential F* | 2457 | Response to light stimulus | Phototransduction |
| 66 | *InR* | *Insulin-like receptor* | 18402 | Adult longevity | Diapause |
| 67 | *lark* | *lark* | 8597 | Circadian rhythm, eclosion | Circadian rhythm |
| 68 | *Mekk1* | *Mekk1* | 7717 | Locomotor activity | Circadian rhythm |
| 69 | *mle* | *maleless* | 11680 | Dosage compensation | Courtship behavior |
| 70 | *Mpk2* | *Mpk2* | 5475 | MAPKKK cascade, response to stress | Circadian rhythm |
| 71 | *na* | *narrow abdomen* | 1517 | Circadian rhythm, adult locomotory behavior | Circadian rhythm |
| 72 | *nan* | *nanchung* | 5842 | Calcium ion transport, sensory perception of sound | Courtship behavior |
| 73 | *Nf1* | *Neurofibromin 1* | 8318 | Regulation of biological process, response to stress | Circadian rhythm |
| 74 | *ninaC* | *neither inactivation nor afterpotential C* | 5125 | Cytoskeleton organization | Phototransduction |
| 75 | *ninaD* | *neither inactivation nor afterpotential D* | 31783 | Rhodopsin biosynthetic process, defense response | Phototransduction |
| 76 | *ninaE* | *neither inactivation nor afterpotential E* | 4550 | Phototransduction | Phototransduction |
| 77 | *nonA* | *no on or off transient A* | 4211 | Male courtship behavior | Courtship behavior |
| 78 | *norpA* | *no receptor potential A* | 3620 | Regulation of biological process | Circadian rhythm |
| 79 | *para* | *paralytic* | 9907 | Response to DDT, response to pyrethroid | Courtship behavior |
| 80 | *PEK* | *Pancreatic eIF-2aplha kinase* | 2087 | Negative regulation of translation | Heat tolerance |
| 81 | *per* | *period* | 2647 | Regulation of biological process, circadian rhythm | Circadian rhythm |
| 82 | *Pgm* | *Phosphogluconate mutase* | 5165 | Glycogen biosynthetic proces | Phototransduction |
| 83 | *Pi3K92E* | *Dp110* | 4141 | Regulation of biological process | Diapause |
| 84 | *Prp5* | *CG6227* | 6227 | Gegulation of alternative nuclear mRNA splicing | Cold tolerance |
| 85 | *pwn* | *pawn* | 11101 | Bristle morphogenesis; phototransduction | Phototransduction |
| 86 | *rdgB* | *retinal degeneration B* | 11111 | Sensory perception of smell | Phototransduction |
| 87 | *Rev1* | *Rev1* | 12189 | Bypass DNA synthesis, DNA repair | Heat tolerance |
| 88 | *Rh3* | *Rhodopsin 3* | 10888 | Phototransduction, UV | Phototransduction |
| 89 | *Rh5* | *Rhodopsin 5* | 5279 | Phototransduction, UV-A | Phototransduction |
| 90 | *RpL11** | *Ribosomal protein L11* | 7726 | Translation, mitotic spindle elongation | Housekeeping gene |
| 91 | *RpL19** | *Ribosomal protein L19* | 2746 | Translation, mitotic spindle elongation | Housekeeping gene |
| 92 | *RpL27A** | *Ribosomal protein L27A* | 15442 | Translation, mitotic spindle elongation | Housekeeping gene |
| 93 | *sgg* | *shaggy* | 2621 | Regulation of biological process | Phototransduction |
| 94 | *Sh* | *Shaker* | 12348 | Locomotory behavior, sleep | Circadian rhythm |
| 95 | *shakB* | *shaking B* | 34358 | Jump response | Phototransduction |
| 96 | *slmb* | *supernumerary limbs* | 3412 | Anatomical structure develop., locomotor behav. | Circadian rhythm |
| 97 | *slo* | *slowpoke* | 10693 | Potassium ion transport, male courtship behavior | Circadian rhythm |
| 98 | *Slob* | *Slowpoke binding protein* | 6772 | Regulation of synaptic transmission | Circadian rhythm |
| 99 | *so* | *sine oculis* | 11121 | Anatomical structure development | Circadian rhythm |
| 100 | *spin* | *spinster* | 8428 | Regulation of developmental process, locomotion | Circadian rhythm |
| 101 | *stv* | *starvin* | 32130 | Apoptosis, proteolysis | Heat tolerance |
| 102 | *syt* | *synaptotagmin* | 3139 | Neurotransmitter secretion, larval locom. behavior | Circadian rhythm |
| 103 | *tilB* | *touch insensitive larva B* | 14620 | Sensory perception of sound, male courtship behav. | Courtship behavior |
| 104 | *tim* | *timeless* | 3234 | Circadian rhythm, sleep | Circadian rhythm |
| 105 | *tipE* | *temperature-induced paralytic E* | 1232 | Sodium ion transport | Courtship behavior |
| 106 | *trp* | *transient receptor potential* | 7875 | Calcium ion transport, response to light stimulus | Phototransduction |
| 107 | *tws* | *twins* | 6235 | Mitotic anaphase | Courtship behavior |
| 108 | *vri* | *vrille* | 14029 | Bristle morphogenesis, locomotor rhythm | Circadian rhythm |

Information of biological processes of the genes is collected from FlyBase database (FB2009_04), released in April 27, 2009. Housekeeping genes are marked with asterisks.
