# Supplementary material for: Changes in gene expression linked with adult reproductive diapause in a northern malt fly species: a candidate gene microarray study
Source: BMC Ecol. 2010 Feb 1;10:3. doi: 10.1186/1472-6785-10-3 (PMC2822739; doi:10.1186/1472-6785-10-3)
Supplement: Additional file 2 — Fold changes in gene expression in microarray/qRT-PCR analysis: comparisons between the three female groups. Fold changes in gene expression of selected candidate genes in microarray and qRT-PCR analysis. Comparisons were made between diapausing, reproducing and young females. Significance levels are indicated as follows: NSNon-significant, *0.05 > P > 0.01, **0.01 > P > 0.001, ***P < 0.001. [file 1472-6785-10-3-S2.DOC]

**Additional file 2.** Fold changes in gene expression in microarray/qRT-PCR analysis: comparisons between the three female groups.

| Gene ID | Biological function | Fold changes and their significance in microarray/qRT-PCR analysis | | | | | |
| --- | --- | --- | --- | --- | --- | --- | --- |
|  |  | Diapausing (D) vs. reproducing (R) females | | Diapausing (D) vs. young (Y) females | | Reproducing (R) vs. young (Y) females | |
|  |  | Upregulation in D females | Upregulation in R females | Upregulation in D females | Upregulation in Y females | Upregulation in R females | Upregulation in Y females |
| *cpo* | Diapause | 1.8^NS^/2.6* |  |  | 3.7*/2.6^NS^ |  | 6.7***/6.4** |
| *FKBP59* | Phototransduction |  | 4.5*/2.1^NS^ |  |  | 2.5*/1.2^NS^ |  |
| *tilB* | Courtship behavior |  | 5.5*/4.1* |  |  | 10.8***/6.8** |  |
| *Fmr1* | Circadian rhythm |  | 3.0**/1.8^NS^ |  |  | 2.2*/1.5* |  |
| *Dca* | Cold tolerance | 4.0***/6.4** |  | 4.1**/2.8^NS^ |  |  |  |
| *Hsp26* | Heat tolerance |  |  |  |  | 30.9***/111*** |  |

Significance levels: ^NS^Non-significant, *0.05>P>0.01, **0.01>P>0.001, ***P<0.001
